# The nuclear gene *rpl18* regulates erythroid maturation via JAK2-STAT3 signaling in zebrafish model of Diamond–Blackfan anemia

**DOI:** 10.1038/s41419-020-2331-5

**Published:** 2020-02-19

**Authors:** Cheng Chen, Mengjia Lu, Shuo Lin, Wei Qin

**Affiliations:** 1 State Key Laboratory of Chemical Oncogenomics, Key Laboratory of Chemical Genomics, Peking University Shenzhen Graduate School, Shenzhen, 518055 China; 2 Department of Molecular, Cell and Developmental Biology, University of California, Los Angeles, CA 90095 USA

**Keywords:** Erythropoiesis, Anaemia

## Abstract

Diamond–Blackfan anemia (DBA) is a rare, inherited bone marrow failure syndrome, characterized by red blood cell aplasia, developmental abnormalities, and enhanced risk of malignancy. However, the underlying pathogenesis of DBA is yet to be understood. Recently, mutations in the gene encoding ribosomal protein (RP) L18 were identified in DBA patients. RPL18 is a crucial component of the ribosomal large subunit but its role in hematopoiesis remains unknown. To genetically model the ribosomal defect identified in DBA, we generated a *rpl18* mutant line in zebrafish, using CRISPR/Cas9 system. Molecular characterization of this mutant line demonstrated that Rpl18 deficiency mirrored the erythroid defects of DBA, namely a lack of mature red blood cells. Rpl18 deficiency caused an increase in p53 activation and JAK2-STAT3 activity. Furthermore, we found inhibitors of JAK2 or STAT3 phosphorylation could rescue anemia in *rpl18* mutants. Our research provides a new in vivo model of Rpl18 deficiency and suggests involvement of signal pathway of JAK2-STAT3 in the DBA pathogenesis.

## Introduction

Diamond–Blackfan anemia (DBA) is an inherited bone marrow failure syndrome characterized by erythroid hypoplasia. It typically presents with macrocytic anemia in infancy, often with normal white blood cell and platelet counts. It is associated with congenital anomalies and a high risk of developing specific cancers, such as acute myeloid leukemia, myelodysplastic syndrome, colon adenocarcinoma, and osteosarcoma^1,2^. Although corticosteroid therapy and bone marrow transplantation are used to treat the patients^3^, significant risks and complications are often associated with these therapies. Therefore, novel therapeutic approaches are needed for treating DBA.

Ribosomal protein (RP) haploinsufficiency is the major cause of DBA and at least 19 genes that affect ribosomal biogenesis have been found to be linked to DBA^4^. Through whole-exome sequencing in patients in DBA, several rare mutations in previously unreported RP genes were found^4,5^, and one such mutation is a novel nonsynonymous mutation (p.L51S) in *rpl18* analysis of pre-rRNA processing in patients’ cells showed an increase in the 36S ribosomal subunit compared with controls, indicating a defect in pre-rRNA processing^6^. However, no in vivo, organismal level research has elucidated the function of *rpl18* in embryonic development.

In this study, an *rpl18*^−/−^ mutant zebrafish line was generated using the CRISPR/Cas9 system to investigate the role of *rpl18* gene in hematopoiesis. Detailed analyses showed that mutation in *rpl18* leads to anemia and severe morphological abnormalities in zebrafish. Inhibition of *p53* partially rescued erythropoiesis in the mutant. RNA-seq analysis showed that the JAK-STAT signaling pathway was abnormally activated in *rpl18* mutant zebrafish. Small molecular inhibitors of STAT3 or JAK2 phosphorylation rescued the anemic phenotype, suggesting that STAT3 and JAK2 could be therapeutic targets for DBA treatment.

## Results

### CRISPR/Cas9 system generates a targeted mutation of *rpl18*^−/−^

**Fig. 1:**
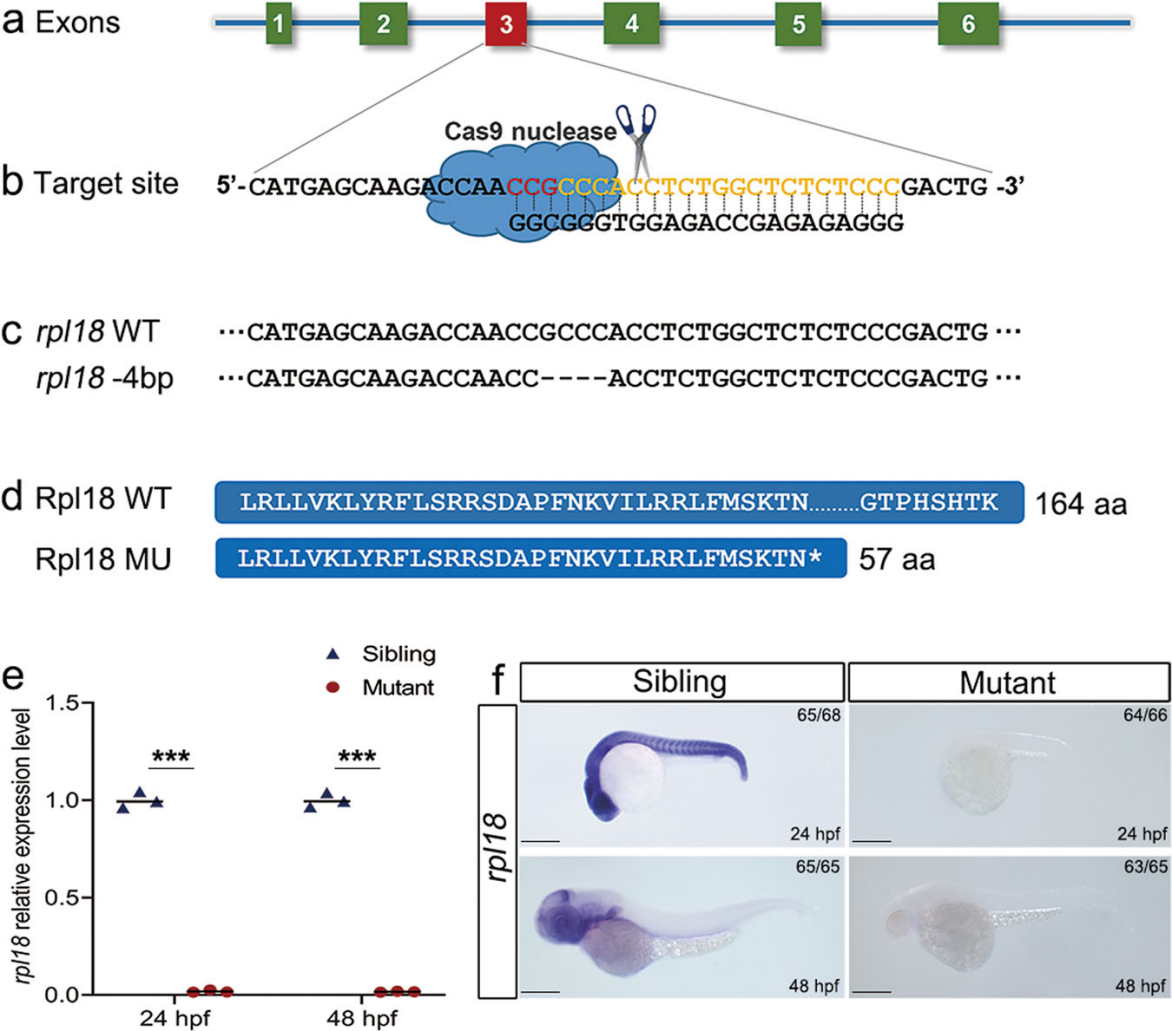
Generation of *rpl18* mutation using CRISPR/Cas9 system. **a**, **b** Illustration describing the target site of *rpl18*. **c**, **d** Genomic sequences and predicted protein sequences based on the DNA sequence in wild-type and *rpl18*^−/−^ mutants. **e** qRT-PCR of *rpl18* transcript levels of wild-type siblings and *rpl18*^−/−^ mutants at 24 and 48 hpf. qRT-PCR experiments were performed biological repeats and technical repeats in triplicate (*N* = 3). ***P < 0.001. **f** WISH results for *rpl18* in siblings and mutants at 24 and 48 hpf, WISH results were verified three times independently (*N* = 3). All scale bars represent 250 μm.

Located on zebrafish chromosome 16, *rpl18* encodes a nucleolar protein, which is highly conserved between human and zebrafish with up to 85% amino acid similarity (Fig. S1). To assess whether *rpl18* is involved in blood cell development in zebrafish, we used CRISPR/Cas9 system to target exon 3 of the gene and generate *rpl18* mutant zebrafish lines (Fig. 1a, b). Injected embryos were raised to adulthood and mutants were screened for germline transmission. Progeny from germline positive carriers were genotyped, and mutations containing a four base pair (bp) deletion were revealed, confirming successful targeting of *rpl18* in zebrafish (Fig. 1c). This mutation led to a truncated Rpl18 protein, which contained only 57 amino acids (Fig. 1d). The PCR sequencing results from wild-type (WT) and homozygous larvae are shown in Fig. S2a, b. Compared with WT larvae, homozygous mutant larvae showed a 4 bp deletion (at GCCC, red frame in Fig. S2a). To verify the reliability of the *rpl18*^−/−^ mutant line, *rpl18* transcripts were measured using quantitative real-time PCR (qRT-PCR) (Fig. 1e). At both 24 and 48 hours post fertilization (hpf), *rpl18* transcripts were significantly downregulated compared with sibling control embryos. Transcript knockdown was further confirmed by whole-mount in situ hybridization (WISH). Strong expression of *rpl18* was observed throughout control embryos at 24 and 48 hpf, however, in our mutants, hardly any *rpl18* expression was observed (Fig. 1f).

### Rpl18 deficiency leads to morphological defects and anemia

To explore the role of *rpl18* in zebrafish development, we analyzed the temporal and spatial expression of *rpl18* at different stages. WISH showed that *rpl18* transcripts were detected as early as the one-cell stage in WT zebrafish embryos (Fig. S3a); *rpl18* continued to be expressed ubiquitously until 36 hpf (Fig. S3b–e). Then, *rpl18* expression became restricted to the eye, head, and pectoral fin bud at 2 days post fertilization (dpf) (Fig. S3f). At 3 dpf, *rpl18* expression was detected in the eye, head, liver, and intestine (Fig. S3g).

**Fig. 2:**
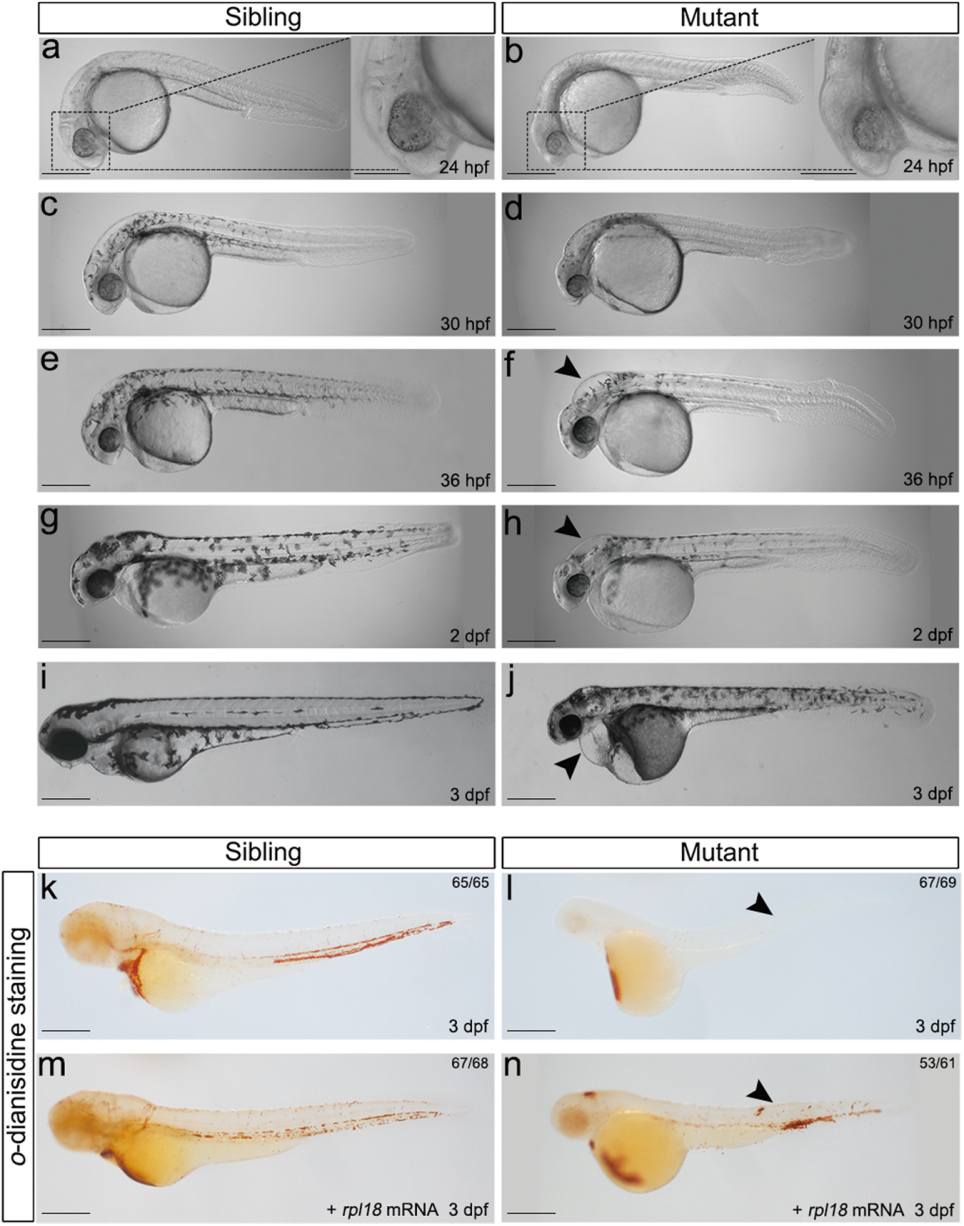
Rpl18-deficient embryos displayed physical abnormalities and anemia. **a**–**j** Phenotypes of wild-type siblings and *rpl18*^−/−^ mutants (*N* = 7). At 24 hpf (**a**, **b**), aplasia of head region (dashed box) can be seen in mutant. At 30 hpf (**c**, **d**), pigmentation was delayed. At 36 hpf (**e**, **f**) and 2 dpf (**g**, **h**), a smaller head and edema (arrowhead) were present. At 3 dpf (**i**, **j**), edema (arrowhead) in heart and shorted tail extension was more apparent. Almost all embryos died at 4 dpf. **k**, **l** *o*-dianisidine staining showed depletion of erythroid cells in *rpl18*^*−/*−^ embryos compared with wild-type siblings (arrowheads pointed to the sites of weak *o*-dianisidine staining, *N* = 5). **m**, **n** *rpl18* mRNA (100 pg/embryo) injected to embryos, staining of 3 dpf mutants with *o*-dianisidine showed a partial recovery (*N* = 3). All scale bars represent 250 μm.

Prior to 22 hpf, no morphological differences were observed between *rpl18* mutant embryos and WT siblings (data not shown); after which, the morphological development of the mutant embryos became gradually abnormal. At 24 hpf, aplasia was seen in the head of Rpl18-deficient embryos (Fig. 2a, b). Small eyes, small heads, and inflation of hindbrain ventricles became apparent between 30 hpf and 2 dpf (Fig. 2c–h). At 3 dpf, the pericardium gradually enlarged and developed edema; additionally, the body length decreased dramatically and the trunk displayed broad apoptosis (Fig. 2i, j). Over time, the severity of the phenotypes increased and the mutation became lethal around 4 dpf.

To investigate the role of *rpl18* in the development of red blood cells in this model, anemia was evaluated by *o*-dianisidine staining. The number of *o*-dianisidine positive cells was greatly reduced in Rpl18-deficient embryos compared with sibling embryos at 3 dpf (Fig. 2k, l), suggesting a role for *rpl18* in the production or maintenance of mature erythroid cells in zebrafish embryos. Moreover, both the morphological defects and lack of erythrocytes were partially rescued in *rpl18* mutant embryos upon co-injection of zebrafish *rpl18* mRNA (100 pg/embryo) (Figs. 2m, n, and S4) but not p.L51S *rpl18* mRNA (data not shown). These data indicate that the p.L51S mutation in RPL18 is likely a pathogenic mutation in patients with DBA.

### Maturation but not specification of erythroid cells was affected in Rpl18-deficient embryos

**Fig. 3:**
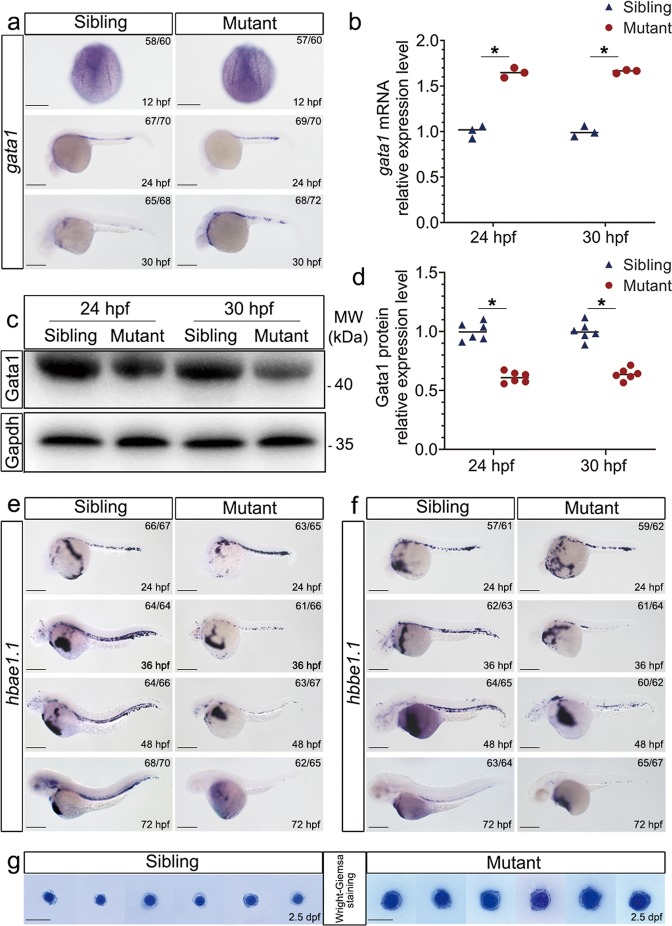
Rpl18 deficiency impaired the erythroid maturation. **a** WISH showed that expression levels of *gata1* in *rpl18*^*−/−*^ embryos was slightly increased at 24 and 30 hpf (*N* = 3). **b** Relative expression of *gata1* mRNA was analyzed by qRT-PCR for *rpl18* siblings and mutants (*N* = 3). *P < 0.05. **c** Western blot (WB) analysis was performed to determine Gata1 protein level. WB with Gata1 antibody demonstrated its reduction in *rpl18*^*−/*−^ mutants at 24 and 30 hpf. **d** The scatter diagram represented quantification of WB. Compared with siblings, the expression of Gata1 protein in mutants decreased by about 40%. The results of three groups of experiments (each experiment was repeated twice) were statistically analyzed, *P < 0.05. WISH showed that expression levels of *hbae1.1* (**e**) and *hbbe1.1* (**f**) in mutants were unaffected at 24 hpf, but reduced significantly from 36 hpf (*N* = 3). Scale bars represent 250 μm. **g** Wright–Giemsa staining of isolated erythroid cells collected from zebrafish embryos at 2.5 dpf. The nucleoli size of erythrocyte in *rpl18* mutants was much larger than siblings (*N* = 4). Scale bars represent 10 μm.

To assess the effect of *rpl18* disruption on erythropoiesis in mutant embryos, we performed WISH for several hematopoietic markers throughout different developmental stages. We found the expression level of *gata1*, one of the earliest markers for specification of erythroid lineage was distinguishable at five-somite stage (Fig. 3a)^7^, suggesting that the specification of erythroid lineage from hematopoietic cells was not affected. Interestingly, the expression level of *gata1* was slightly increased at 24 and 30 hpf (Fig. 3a), qRT-PCR result further confirmed this phenomenon (Fig. 3b). However, the levels of Gata1 protein was reduced in *rpl18*^*−/−*^ mutant (Fig. 3c, d), which is consistent with previous study^8,9^. This finding suggested that there might be some mechanism promoting the mRNA expression of *gata1* but impairing the translation of Gata1 protein in Rpl18-deficient embryos. Then we investigated the expression pattern of two globin transcripts *hbae1.1* and *hbbe1.1*. There was a similar expression level of global transcript at 24 hpf, but the expression of *hbae1.1* and *hbbe1.1* appeared dramatically decreased from 36 to 72 hpf (Fig. 3e, f). Meanwhile, the expression level of *lyz* and *mpx* was distinguishable at 30 and 48 hpf (Fig. S5a–e). Next, we analyzed the status of erythrocytes. Evidently, erythroid cells in *rpl18* mutant arrested at the basophilic stage at this time point (Fig. 3g), suggesting the maturation of erythrocyte was impaired in *rpl18* mutant. Overall, these findings demonstrated that erythropoiesis was specifically affected in *rpl18* mutants and the anemia was due to a late-stage terminal maturation of erythroid cells.

### Transcriptome analysis of Rpl18-deficient embryos

**Fig. 4:**
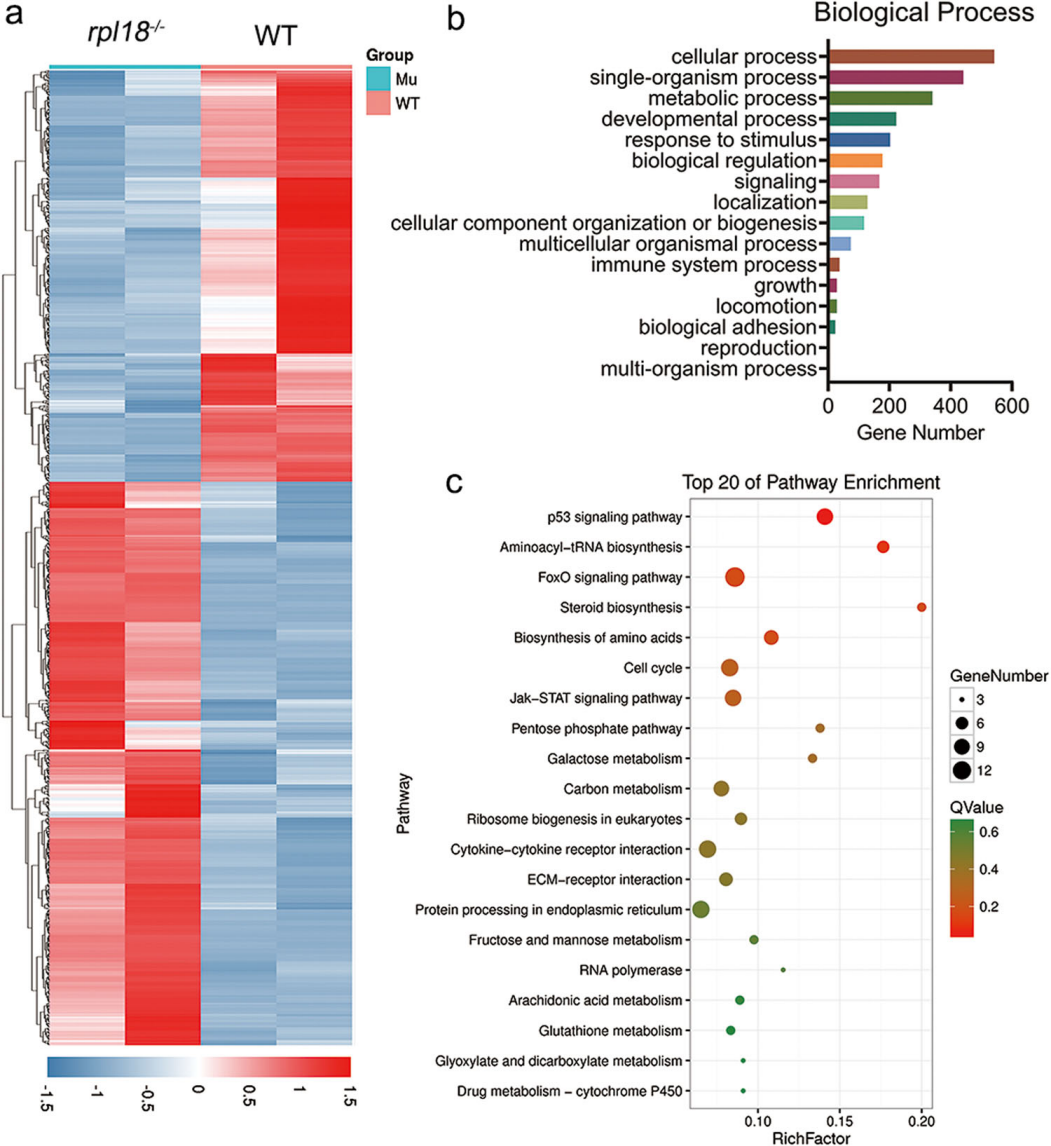
Differential gene expression profile, gene ontology, and pathway enrichment analysis for *rpl18* mutants and siblings. **a** The heat map of the differential gene expression profile for *rpl18*^−*/*−^ embryos and siblings assessed by transcriptome sequencing. **b** The gene ontology (GO) enrichment analysis of biological process indicated that differentially expressed genes occurred in these processes. **c** Pathway enrichment analysis identified the top 20 of pathway affected in mutants.

Previous studies have shown that RP deficiency leads to the accumulation of nonprocessed pre-rRNA and impaired ribosome biogenesis^10^. To better understand the mechanism of how *rpl18* affects zebrafish development, we used RNA-seq to analyze global transcriptome changes in Rpl18*-*deficient embryos. There were 663 upregulated genes and 484 downregulated genes in *rpl18* mutants compared with the control sample (fold change, 2.0; *P* value, 0.05) (Fig. 4a). Based on GO annotation, the differentially expressed genes were enriched in the GO terms of cellular process, single organism process, metabolic process, response to stimulus, and immune system process, among others (Fig. 4b). KEGG pathway analysis showed that the p53 signaling pathway, cell cycle, JAK-STAT signaling pathway, and ribosome biogenesis were significantly disrupted in the *rpl18* mutants (Fig. 4c). These data confirm that loss of *rpl18* does indeed affect ribosome-related gene expression.

### Delayed in erythroid maturation is p53 dependent

**Fig. 5:**
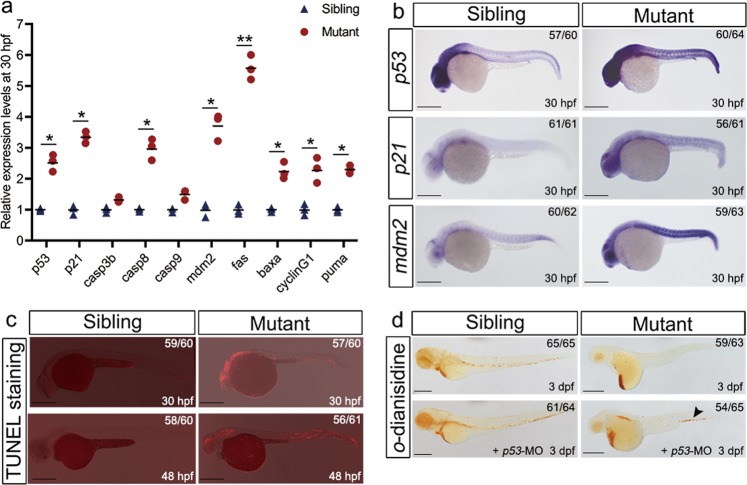
Upregulation of p53 activity partially contributed to the erythroid phenotype in *rpl18* mutants. **a** Transcript quantification of *p53* and *p53* related genes were verified by qRT-PCR. All the *p53* related genes were highly expressed in *rpl18* mutants at 30 hpf (*N* = 3), *P < 0.05. **P < 0.01. **b** WISH showed that *p53, p21*, and *mdm2* expression was elevated in *rpl18* mutants at 30 hpf (*N* = 3). **c** In situ cell death staining result showed that the signal of apoptosis can be observed thought the body at 30 and 48 hpf (*N* = 4). **d** An increase of hemoglobin level appeared in mutants at 3 dpf after *p53* knockdown (*N* = 3). Arrowhead points to the recovery of erythrocytes. All scale bars represent 250 μm.

Ribosome stress has been shown to cause elevated levels of p53 and cell cycle arrest^11–13^. qRT-PCR analysis (Fig. 5a) and WISH data (Fig. 5b) showed that *p53* and *p53* target genes were increased in Rpl18-deficient embryos. Increased apoptosis was detected via TUNEL staining at 30 and 48 hpf throughout *rpl18* mutant embryos (Fig. 5c), suggesting that morphological defects may be partially due to increased apoptosis. To determine whether the defects in *rpl18* mutants were p53 dependent, we injected *p53* morpholinos into Rpl18-deficient embryos. Knockdown of *p53* partially rescued both the morphological defects and anemic phenotype at 3 dpf (Figs. 5d, and S6). Taken together, these findings suggest that p53-dependent pathways are upregulated and involved in the anemic phenotype in *rpl18* mutant zebrafish.

### Increased STAT3 phosphorylation impaired erythroid cell development

**Fig. 6:**
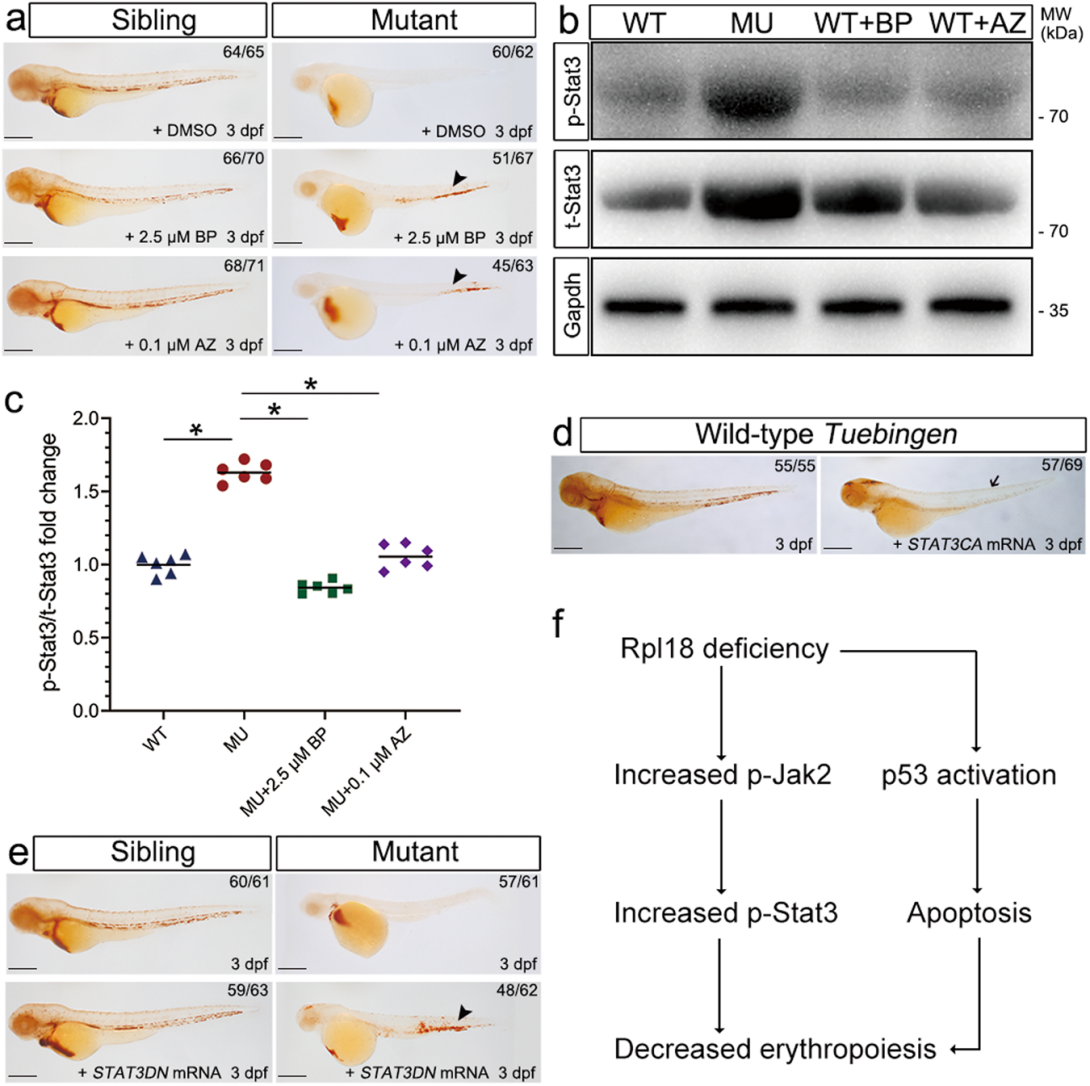
Inhibition of the phosphorylation of Jak2 or Stat3 rescued hemoglobin level in *rpl18* mutants. **a** *rpl18* sibling and mutant embryos were treated with BP (T3708, STAT3 inhibitor) or AZ (T6309, JAK2 inhibitor) (*N* = 5). At 3 dpf, *o*-dianisidine staining of mutant embryos showed obvious recovery with BP (2.5 μM) treatment, and mutants treated with AZ (0.1 μM) also be seen partially restored (arrowhead). **b** The total amounts of Stat3 (t-Stat3) and the phosphorylation status of endogenous Stat3 protein (p-Stat3) was evaluated in *rpl18* siblings and mutants. The protein levels of t-Stat3 and p-Stat3 had a significant increase in mutants, compared with wild-type (WT) embryos at 30 hpf. Both the t-Stat3 and p-Stat3 were dramatically decreased in Rpl18-deficient embryos treated with BP or AZ separately, as shown by western blotting. **c** The scatter diagram represented quantification of western blots. The results of three groups of experiments (twice technical repeated for each group) were statistically analyzed, *P < 0.05. **d** The *o*-dianisidine staining displayed that most of erythroid cells disappeared (arrow) in wild-type embryos after injection the *STAT3CA* (constitutively active STAT3 with the double mutation A661C N663C) mRNA, compared with sibling embryos at 3 dpf (*N* = 3). **e** Erythrocytes were partially rescued in *rpl18* mutants that had been injected with *STAT3DN* (dominant-negative STAT3) mRNA at 3 dpf (*N* = 3). Black arrowhead points to the recovery of erythrocytes. **f** Model of the defective erythroid maturation in Rpl18 deficiency through increased phosphorylated Stat3 expression. All scale bars represent 250 μm.

Previous studies reported that overexpression of phosphorylated STAT3 has an inhibitory effect on erythroid cell maturation^14,15^. Our RNA-seq data showed that the JAK-STAT signaling pathway was abnormally activated in Rpl18-deficient embryos (Fig. 4c). More importantly, expression of JAK-STAT pathway-related genes, such as *il6, il6st, stat3, irf9, socs3a*, and *socs3b*, were confirmed to be elevated by qRT-PCR (Fig. S7a, b). To investigate whether this pathway and which pathway component affects the maturation of erythroid cells in our DBA model, *rpl18* mutant zebrafish embryos were exposed to several small molecular inhibitors targeting different components of the JAK-STAT pathway. We found that three specific inhibitors of STAT3 phosphorylation (Figs. 6a, and S8c–f) and two specific inhibitors of JAK2 phosphorylation (Figs. 6a, and S8k, l) rescued, to various degrees, the anemic but not the morphological phenotype at 3 dpf. No rescue was observed in response to the use of other targeted inhibitors (Figs. S8g–j, and S8m–t). Western blot results further confirmed that inhibition of phosphorylated STAT3 or phosphorylated JAK2 were associated with rescue of the anemic phenotype in our *rpl18* mutant DBA model (Fig. 6b, c). To further test the mechanism at a molecular level, constitutively active human *STAT3* mRNA was injected into TU embryos. Upon overexpression of constitutively active *STAT3* mRNA, *o*-dianisidine staining revealed reduced hemoglobin at 3 dpf (Fig. 6d), suggesting that overphosphorylation of STAT3 is sufficient to impair erythroid cell development. In addition, injection of dominant-negative human *STAT3* mRNA into Rpl18-deficient embryos largely rescued the number of *o*-dianisidine-positive erythroid cells (Fig. 6e). Taken together, we conclude that deficiency of *rpl18* likely induces defect in erythroid cell maturation through JAK2-STAT3 signaling (Fig. 6f).

## Discussion

DBA has been studied for several decades and, although mutations in many RPs have been reported to cause this disease, there are still some unidentified pathogenic variants in DBA patients. In this work, we successfully mutagenized a newly identified locus, *rpl18*, in zebrafish using CRISPR/Cas9 gene-editing technology. Rpl18 deficiency caused gross morphological defects and an anemic phenotype, which is consistent with previous reports in zebrafish. Not surprisingly, *p53* and its downstream genes were elevated in *rpl18* mutants. Inhibition of p53 activity using *p53* morpholinos only partially rescued both the morphological defects and anemic phenotype, suggesting that other factors or pathways are likely involved in pathogenesis of *rpl18*-induced DBA.

Morphological and erythroid defects can be ameliorated in Rps19-deficient zebrafish embryos treated with L-leucine or exogenous nucleosides^16,17^, but no recognizable improvements were observed in *rpl18* mutants in response to these treatments (data not shown). Danilova et al. reported that the innate immune system, including interferon and inflammatory cytokines, was activated in Rps19-deficient zebrafish and RPS19-deficient human primary cells^18^. The expression of related genes, such as *tlr9*, was also increased in Rpl18-deficient embryos (Fig. S9). We observed that the JAK-STAT pathway was significantly activated in our DBA model. Upon analyzing the RNA-seq data, this same phenomenon occurred in the *rps19* mutant zebrafish DBA model^19^. Either inhibition of STAT3 or JAK2 phosphorylation partially rescued the anemic phenotype but not the morphological defects in *rpl18* mutant zebrafish, suggesting that the role of the JAK2-STAT3 pathway is specific to the maturation of erythroid cells. Like in *rpl18* mutants, restoration of hemoglobin levels was observed upon inhibition of STAT3 phosphorylation in *rps19* morphant zebrafishes (Fig. S10), suggesting that overphosphorylation of STAT3 may be a phenomenon common to different genetic models of DBA.

Previous studies have shown that phosphorylated STAT3 inhibits GATA1 through a direct protein–protein interaction, and GATA1 can reverse STAT3-mediated γ-globin gene silencing in human erythroid cells; in our *rpl18* mutant DBA model, a slight increase in *gata1* mRNA expression but reduced levels of Gata1 protein was observed at 24 and 30 hpf. Therefore, a positive feedback mechanism between phosphorylated STAT3 and GATA1 may exist, where overphosphorylation of STAT3 promotes *gata1* mRNA expression but meanwhile inhibits the translation of GATA1 protein. Further experiments are required to test these hypotheses.

Haploinsufficiency of RPs has been implicated in several bone marrow failure disorders including DBA^20,21^. The first animal model of DBA was generated in mouse. But unlike DBA patients, heterozygous deletion of *rps19* did not have a hematopoietic phenotype, and the homozygous mouse was embryonic lethal^22^. The similar phenomenon was observed in *rps19* mutant zebrafish and other known DBA-related knockout fish^23,24^. But in our *rpl18*-induced DBA model, we observed a noteworthy phenomenon. Generally, we should get two-thirds of heterozygous *rpl18*^+/−^ offspring from in cross of two heterozygous parents, but we could only get one-fifth *rpl18*^+/−^ fish (Table S1), which suggests that some heterozygous fishes could have died before we screened. In addition, the identified heterozygous fish had a shorter life span (5 months), which means heterozygous zebrafish with the same genotype (*rpl18*^+/−^) has different phenotype. This phenomenon has been noted for over five generations. In patients, why individuals with the same mutation can present with different symptoms or different severity of disease is still unknown^25^. Here we evaluated the erythroid production in the heterozygous mutants. Quantitative analysis of RBC mass showed that the different heterozygous adults had different and less hemoglobin concentration than WT. Moreover, Wright–Giemsa staining analysis indicated that heterozygous mutants have more immature erythrocytes compared with the WT (Fig. S11b). These results demonstrated that in zebrafish, the same mutation can also result in different severity of the disease, which mimics the symptoms of the DBA in patients.

To the best of our knowledge, this is the first demonstration that the anemic defect in a zebrafish DBA model can be rescued by inhibition of STAT3 or JAK2 phosphorylation. This finding enhances our understanding of the pathogenesis of the disease. It is worth noting that one drug targeting STAT3 that is in clinical development, ochromycinone (STA-21)^26^, can attenuate the anemic phenotype in our zebrafish DBA model (Fig. S12). Based on our findings, therapies targeting JAK2-STAT3 signaling should be further explored as potential treatments for DBA.

## Materials and methods

### Zebrafish maintenance

Zebrafish was raised and maintained under standard laboratory conditions at 28.5 °C. WT TU zebrafish were used in this study. All zebrafish experiments were approved by Institutional Animal Care and Use Committee of Peking University. All zebrafish embryos or adults used in this study were chosen randomly. Embryos or adults were genotyped after analysis.

### Cas9 mRNA and gRNA synthesis

For making zCas9 mRNA, the template was linearized by XbaI (pT3TS-zCas9) digestion^27^ and purified using TIANquik mini purification kit (TIANGEN). Capped zCas9 mRNA was synthesized using T3 mMESSAGE mMACHINE kit (Ambion) and purified using an RNeasy FFPE kit (Qiagen). All sgRNAs (single-guide RNAs) templates in this study were prepared according to the cloning-independent sgRNA generation method^28^. All gRNAs were transcribed in vitro using the T7 RNA Polymerase (TaKaRa), and purified using RNeasy^®^ FFPE Kit (Qiagen). The size and quality of resulting mRNA and sgRNAs was confirmed by electrophoresis through a 1.5% agarose gel.

### Generation of the *rpl18*^−/−^ homozygous mutants

A solution (1–2 nl) containing zCas9 mRNA (200 ng/μl) and sgRNA (30 ng/μl) was co-injected into one-cell-stage zebrafish embryos. These embryos were incubated at 28.5 °C for observation of phenotypes and PCR amplification. Injected embryos were raised as F0 to adulthood. F0 adult zebrafish was outcrossed to a WT fish (*Tuebingen*) and offspring embryos were collected. The region flanking of the target site of offspring embryos were PCR amplified using forward primer: 5′-ATGCGTCAGTTTTGGCCAGT-3′ and reverse primer: 5′-TGTGGACGGAGAGCTTTTCA-3′. The purified PCR product was made by TA cloning using pEASY-T1 Simple Cloning Kit (TransGen) and then sequencing. Offspring of germline transmitted F0 zebrafishes were raised as F1 to adulthood. To identify germline transmitted F1 fishes, the tail of sexually mature F1 was cut off to obtain the genome, which was then genotyped and sequencing using forward primer: 5′-ACACCTCTGTGCTTTTTGTGC-3′ and reverse primer: 5′-ACATACCCTGTCTCCCTTGC-3′. Pairs of *rpl18*^*+/*−^ F1 heterozygotes incrossed and generated the *rpl18*^*−/*−^ homozygous mutants.

### Constructs

The zebrafish *rpl18* gene were amplified and subcloned into vector PCS2+ for mRNA synthesis following NotI (NEB) digestion. Human STAT3DN (a dominant-negative STAT3), STAT3CA (constitutively active STAT3 with the double mutation A661C N663C) constructs have been described previously^29^. The sequences of *p53* MO was descried by Langherinrich et al.^30^.

### qRT-PCR

Total RNA was isolated from whole embryos (30–35 embryos per group) at specific stages, using TRIzol^®^ Reagent (Life Technologies), and following the manufacture’s protocol. The cDNA was reverse transcribed from 1 μg total RNA using PrimerScriptTM RT reagent kit (TaKaRa) for next real-time quantitative PCR analysis. All quantitative PCR was performed biological repeat in triplicates (each biological repeat contains three technical repeats) using TB Green^®^ Premix Ex Taq™ (TaKaRa) and the CFX96 real-time PCR detection system (Bio-Rad). The used PCR primer sequences are listed in Table S2.

### RNA-seq analysis

Two independent batches of samples were collected for RNA-seq. The first group between mutants and siblings, total RNA was isolated from whole embryos (70–80 embryos per group) at 28 hpf. The Second group, total RNA was extracted at 30 hpf. TRIzol^®^ Reagent (Life Technologies) was used.

RNA concentration and purity were measured by nanodrop2000 (Thermo). Total RNA concentrations ≥ 200 μg/μl and the purity RIN ≥ 7, 28S/18S ≥ 1.0. The mRNA was enriched by Oligo (dT) beads, then the enriched mRNA was fragmented into short fragments (200–700 nt) and reverse synthesized into first-strand cDNA using random primers. The second-strand cDNA was performed end reparation, poly-A addition and Illumina sequencing adapters ligation following purification with QiaQuick PCR extraction kit. After the PCR amplification, these products were sequenced by Illumina HiSeqTM.

For bioinformatics analysis, raw reads containing adapter, low quality reads (*Q* value ≤ 20) and reads containing unknown nucleotides (more than 10%) were removed. Then the RNA removed reads mapped to the zebrafish (Danio Rerio) reference genome by TopHat2 (version 2.0.3.12)^31^. Differentially expressed genes were analyzed by edgeR package (fold change > 2, FDR < 0.05) in a comparison as significant DEGs^31^. DEGs were subjected to enrichment analysis of gene ontology and pathway. For GO analysis, all DEGs were mapped to GO terms in the Gene Ontology database, and gene numbers were calculated for every term^32^. For pathway enrichment, KEGG was the major public pathway-related database. The calculated *P* value was execute FDR correction (FDR ≤ 0.05 as a threshold)^33^.

### WISH

Embryos of different periods were fixed in 4% paraformaldehyde (PFA) solution overnight at 4 °C. After fixation and gradient dehydration, embryos can store at −20 °C for long time. Linearized plasmids were employed as templates to synthesize digoxigenin-labeled antisense probes using the DIG RNA labeling mix (Roche) and T7 RNA Polymerase (TaKaRa). After synthesis, these probes were purified by the RNeasy^®^ FFPE Kit (Qiagen). Finally, WISH was performed as described using probes including *rpl18*, *gata1*, *hbae1.1*, *hbbe1.1*, *lyz*, *mpx*, *p53*, *p21*, and *mdm2*. The stained embryos were photographed using a microscope (ZEISS, Imager.A1).

### Genotype identification

For genotyping, the genome was extracted from the embryos when imaging was finished. Generally, an embryo was placed in a centrifuge tube with immersing in 10 μl of 50 mM NaOH and was incubated at 95 °C for 20 min twice. After vortexing, 1 μl of 1 M Tris-HCl (pH = 8.0) was added to the previous solution to mix well. Finally, the prepared DNA was amplified for sequencing to identify genotypes.

### Protein extraction and western bolt

Thirty to thirty-five whole embryos at 30 hpf were collected and lysed in M-PER™ mammalian protein extraction reagent (Thermo) that had been mixed with protease and phosphatase inhibitors. Protein extraction experiment needs to be done on ice. Protein concentration was measured with Pierce^®^ BCA protein assay reagent (Thermo). Ten to fifteen milligrams of total protein was used for SDS-PAGE (Tris glycine) electrophoresis, and the next procedure of western blot need to refer to protocols of different antibodies. The following antibodies for zebrafish were used in this project: GAPDH (GeneTex), GATA1 (GeneTex), STAT3 (Abcam), anti-phospho-STAT3 (Tyr708) (MBL), anti-mouse IgG HRP-linked antibody (CST), and anti-rabbit IgG HRP-linked antibody (CST). Imaging results were quantified using ImageJ software.

### *o*-Dianisidine staining

Embryos were fixed at 4 °C using 4% PFA followed by four washes (5 min each) in 1X PBST. Then embryos were dyed using *o*-dianisidine staining solution (40% anhydrous ethanol, 0.65% hydrogen peroxide, 10 mM sodium acetate, and 0.6 mg/ml *o*-dianisidine [Sigma]) in the dark for 50 min. After incubation, embryos were washed four times (5 min each) in 1X PBST and then added into the bleach solution (1% potassium hydroxide, 3% hydrogen peroxide) until the pigmentation was removed. Embryos were then washed in 1X PBST and imaged by microscope (ZEISS, Imager.A1).

### TUNEL staining

Thirty and forty-eight hours post fertilization embryos were fixed with 4% PFA overnight at 4 °C followed by five washes (5 min each) in 1X PBST. Then embryos were treated with acetone for 5 min and digested with proteinase K (Roche) (10 μg/ml) at room temperature for 15–20 min. After each of these treatments, 1X PBST washing was required for next step. TUNEL staining was performed using the in situ Cell Death Detection Kit and TMR Red (Roche). The reaction was stopped after 1.5 h dark treatment and immediately imaged. The pictures were captured by a fluorescence microscope (ZEISS, Discovery.V2.0).

### Wright–Giemsa staining

Zebrafish embryos at 60 hpf were anesthetized by tricaine, then were immersed in mixture solution (40% FBS [HyClone] and a final concentration of 5 mM EDTA solution in 1X PBS). Red blood cells were collected from the heart using a microinjection needle. The cell solution was centrifuged at 1000 rpm for 5 min and the supernatant was discarded. Then the enriched cells were smeared evenly on slide and air-dried rapidly. After fixing in the methanol for 5 min, the slide was soaked in rapid Wright–Giemsa staining solution (BBI Life Sciences) for 10 min. Finally, slides were rinsed with deionized water. Images were captured with 100× lens after air dry. Assessment of erythrocyte status was based on previously published article^34^.

### Hemoglobin quantification

Peripheral blood was collected from anaesthetized WT and *rpl18*^+/−^ heterozygous zebrafish (4 months old, both male and female) by cutting the tail^35^. To prevent clotting, pipette tips and scissors were treated with 1% heparin solution in advance. Hemoglobin was measured with the Hemoglobin Assay Kit (Sigma-Aldrich, MAK115) according to the manufacturer’s protocol.

### Inhibitor treatments

Most of inhibitors used were purchased from TargetMol, including T3708 (BP-1-102), T6309 (AZ960), T2814 (cryptotanshinone), T4234 (HJC0152 hydrochloride), T4216 (STAT5 inhibitor), T1038 (fludarabine), T1849 (momelotinib), T6122 (CEP-33779), T3620 (solcitinib), T3998 (itacitinib), and T4657 (WHI-P97). Only ochromycinone (STA-21) were purchased from Selleck. All inhibitors were dissolved in DMSO to a storage concentration of 10 mM and stored at −80 °C. It is important to avoid repeated freezing and thawing for inhibitor activity. Embryos from the same parent were divided into control and treatment groups at 6 hpf. Every treatment group was raised in 1X Holt buffer with all kinds of inhibitors and control groups was raised in in 1X Holt buffer. Embryos were observed and collected for the following experiments at 3 dpf. Each inhibitor treatment has at least three biological replications and three technical replications.

### Statistics and reproducibility

Experiments were biologically repeated at least three times independently, and the number of times an experiment was repeated (*N*) had stated in the legend. All statistical data were calculated and graphed using GraphPad Prism 8 Software. Then, the mean and standard deviation were calculated. For two groups’ comparison, *P* values were calculated using two-tailed unpaired Student’s *t* test and less than 0.05 was considered as significant.

## Supplementary information

Reproducibility Checklist

Supplementary Table Information

Supplementary Figure Legends

Supplementary Figure 1

Supplementary Figure 2

Supplementary Figure 3

Supplementary Figure 4

Supplementary Figure 5

Supplementary Figure 6

Supplementary Figure 7

Supplementary Figure 8

Supplementary Figure 9

Supplementary Figure 10

Supplementary Figure 11

Supplementary Figure 12

## Data Availability

All data generated or analyzed during this study are included in this article and its supplementary information files. The raw RNA-seq data was uploaded to NCBI SRA database. The SRA accession number: PRJNA597556.
