# Supplementary material for: The nuclear gene rpl18 regulates erythroid maturation via JAK2-STAT3 signaling in zebrafish model of Diamond–Blackfan anemia
Source: Cell Death Dis. 2020 Feb 19;11(2):135. doi: 10.1038/s41419-020-2331-5 (PMC7031319; doi:10.1038/s41419-020-2331-5)
Supplement: Supplementary file 2 — Supplementary Table Information [file 41419_2020_2331_MOESM2_ESM.docx]

Supplemental Material

Supplementary Table Information

Table S1. Generation efficiency of the *rpl18^+/-^* heterozygous

| Generations | Total numbers of fish screened | *rpl18^+/-^* heterozygous |
| --- | --- | --- |
| 1 | 32 | 6（18.8%） |
| 2 | 64 | 13（20.3%） |
| 3 | 48 | 10（20.8%） |
| 4 | 24 | 4（20.8%） |
| 5 | 100 | 21（21.0%） |

Table S2. Primer sequences for quantitative real-time PCR analysis

| Genes | Forward primer | Reverse primer |
| --- | --- | --- |
| *gapdh* | TCCAGTACGACTCCACCCAT | TGACTCTCTTTGCACCACCC |
| *rpl18* | TGGCTCTCTCCCGACTGATT | ATGCCTGTACACCTGTCTGC |
| *p53* | ACAGCTTGGTGCTGAATGGA | TTCAGCAGTGTAGCAGCAAAC |
| *p21* | AGCTTCGACTTTGCGTCTGA | CCACTAGACGCTTCTTGGCT |
| *casp3b* | GTAAGTCGGCCATGTTTGCG | CTCGTCACCTACACCGTCAC |
| *casp8* | CTGCTCAAACGAACAGGCAC | AAACTGTGCCCTTCTCTCCG |
| *casp9* | TCCAACATCGACTGCGACAA | CCGTCAACACCATGAACTGC |
| *mdm2* | GCGAGTCTTCGGATGCAAAC | CTGTGAATCCTGCCTCGGTT |
| *fas* | TATAGCTGGCGCTTGAGACG | TCACAAAAGCGACCCTCCTC |
| *baxa* | TACTTTGCCTGTCGCCTTGT | CAGCGAGGAAAACTCCGACT |
| *cyclinG1* | CATCTCTAAAAGAGGCTCTAGATGG | CACACAAACCAGGTCTCCAG |
| *puma* | TGGAAAGCAGAGTGGACGAA | GATGGCAGGGCTGGATGA |
| *stat3* | TTCATCTGTGTCACCCCGTG | GGCAGGTGTCCATATCCGAG |
| *il6* | TTCATGAGTCTCGCTGACCC | GAGGAGTGCTGATCCTGACC |
| *il6r* | CAGGGTGACACGGGGAATC | GTCCATTCACAGCGGACCTTA |
| *il6st* | CACGCCTGGGAAAAGAAACG | GACCCTCCGGCTGAATTGAA |
| *il10* | TGGAGACCATTCTGCCAACA | AGAGCAAATCAAGCTCCCCC |
| *jak2a* | CTAGCATGGGCGCTACATCA | TCCGCAGTAGATCTCCCACA |
| *jak2b* | CCGAGCAGGTCACTATCGTC | GTGATGGGCATCTGTGGTCA |
| *irf9* | AGGTCACAGAAAGGTCACAGC | GCCTGTGGTGCAACAATCTC |
| *irf7* | GCATGCAGTTTCCCAGTCAC | GGAAGCGTATTTGCTCCCCT |
| *bcl-xl* | ACGCATCGCAGAATGGATGA | TTCTGTGCAATGAGTCCCCC |
| *pim1* | ATGGGGAGAACTGCCCAATG | CTTTGATGTCGCGGTGAACG |
| *mcl1a* | AACTCCATCACGCCATACCG | TGCTGGAGCTCCTTCAATCG |
| *mcl1b* | AACTGGGGTCGTATTGCCAG | TGTGTTTCTCACAGCTGCCT |
| *cmyc* | GCAAGGTTGCATCACCACAG | GACTGGGGTACCTCGACTCT |
| *socs3a* | CCAAGAGCATGCAATCCGTG | CTCTTGCTGGACACATCCGT |
| *socs3b* | TTTTCCTGCAGACTGACCCG | CCGTTTGGTGGACACGTCTAT |
| *mpx* | ATGCTCACCATCTTTGGGCA | GCAAAGACGTTCGCAATGGT |
| *lyz* | CGTGGATGTCCTCGTGTGAA | CTGAACAGGCCACTTTGCAC |
| *gata1* | ACTGCCACCCGTTGATGTAG | TACTAGTGTGGCAGTTGGCG |
