# Supplementary material for: The nuclear gene rpl18 regulates erythroid maturation via JAK2-STAT3 signaling in zebrafish model of Diamond–Blackfan anemia
Source: Cell Death Dis. 2020 Feb 19;11(2):135. doi: 10.1038/s41419-020-2331-5 (PMC7031319; doi:10.1038/s41419-020-2331-5)
Supplement: Supplementary file 3 — Supplementary Figure Legends [file 41419_2020_2331_MOESM3_ESM.docx]

Supplemental Material

Supplementary Figure Legends

Supplementary Figure S1. Rpl18 protein alignment between human and zebrafish. Leucine at position 51 (arrowhead) was highly conserved between zebrafish and human. CLUSTAL multiple sequence alignment by MUSCLE (3.8). Black arrowhead points to the pathogenic site in human DBA patients.

Supplementary Figure S2. Sequencing results of *rpl18* sibling and mutant (N=10). There were sequencing maps of wild-type (a) and homozygous (b) zebrafish. The sequence in red frame was the deleted base pairs in *rpl18* mutant.

Supplementary Figure S3. Temporal and spatial expression of the *rpl18* gene during early zebrafish development. (a) WISH with *rpl18* probe indicated maternal expression in one-cell. (b-e) Subsequently, it was widely expressed throughout the embryo. (f-g) *rpl18* mRNA was intensively expressed in the head region during 48 hpf and 72 hpf. All scale bars represent 250 μm, N=3.

Supplementary Figure S4. *rpl18* mutant morphology was obviously recovered by injecting *rpl18* mRNA (N=3), especially the eyes and the heart (arrowhead). All scale bars represent 250 μm.

Supplementary Figure S5. Myeloid cells were not significantly affected by Rpl18 deficiency. (a-d) At 48 hpf, in situ hybridization of *mpx*, *lyz* had similar expression parterns between *rpl18^-/-^* embroys and siblings (N=3). (e) qRT-PCR results of *mpx* and *lyz* at the same stages (N=3).

Supplementary Figure S6. Slight morphological rescue (arrowhead) was observed in mutants with knockdown of *p53* (N=3). All scale bars represent 250 μm.

Supplementary Figure S7. mRNA expression levels of JAK-STAT pathway related genes were verified by qRT-PCR. (a) *stat3* mRNA was highly expressed following Rpl18 deficiency from 30 hpf to 72 hpf (N=3). **,P<0.01. (b) Most of JAK-STAT pathway related genes expression level increased in the mutants compared to sibling embryos (N=3). *, P<0.05. **,P<0.01.

Supplementary Figure S8. *rpl18* mutants and siblings were treated with different JAK-STAT related inhibitors (N=5). After treatment with each inhibitor, the *rpl18* mutants and siblings at 3 dpf were collected for *o*-dianisidine staining. (a-b) *rpl18* siblings and mutants were treated with DMSO as control. (c-f) In addition to BP, two other effective STAT3 inhibitors were found to restore erythrocytes (arrowhead). STAT1 inhibitor (g-h) and STAT5 inhibitor (i-j) had not been shown to improve anemia caused by *rpl18* deficiency. (k-l) Except for AZ, there was another JAK2 inhibitor that also increaseed erythrocytes. But the results showed that JAK1 inhibitors (m-p), JAK3 inhibitor (q-r) and JAK1/2/3 inhibitor (s-t) did not rescue anemia in *rpl18* deficiency embroys at 3 dpf. All scale bars represent 250 μm.

Supplementary Figure S9. Expression levels of genes associated with immune system were veried by qRT-PCR (N=3). **, P<0.01. ***, P<0.001.

Supplementary Figure S10. Hemoglobin level in *rps19* morphants was increased by treating with BP (arrowhead) (N=3). All scale bars represent 250 μm.

Supplementary Figure S11. *rpl18* heterozygous zebrafish adults had decreased hemoglobin concentration and immature erythrocytes. Peripheral blood was collected from wild-type and heterozygous zebrafish adult (four months old). (a) Hemoglobin concentration was measured with the Hemoglobin Assay Kit. Quantitative analysis of hemoglobin levels indicated that wild-type (11.64 ± 1.21 g/dl, N=8) adults contain up to 2.3 times as much hemoglobin as *rpl18^+/-^* heterozygous adults (5.01 ± 1.18 g/dl, N=8). Values of hemoglobin concentration expressed in mean ± standard deviation. *, P<0.05. (b) Wright-Giemsa staining results showed that heterozygous adults contained more immature erythrocytes (red arrowheads) compared with wild-type siblings (N=8). Scale bars represent 20 μm.

Supplementary Figure S12. Erythrocytes were partially rescued in *rpl18* mutants that had been treated with Ochromycinone (STA-21) at 3 dpf (N=3). Black arrowhead shows the recovery of erythrocytes. All scale bars represent 250 μm.
