# Supplementary figures and images for: The nuclear gene rpl18 regulates erythroid maturation via JAK2-STAT3 signaling in zebrafish model of Diamond–Blackfan anemia
Source: Cell Death Dis. 2020 Feb 19;11(2):135. doi: 10.1038/s41419-020-2331-5 (PMC7031319; doi:10.1038/s41419-020-2331-5)

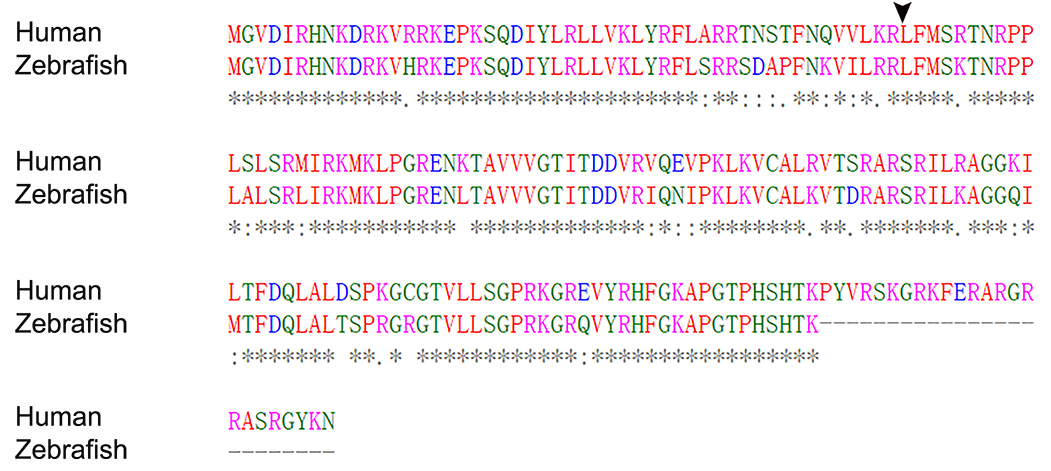

Supplement: Supplementary file 4 — Supplementary Figure 1 [file 41419_2020_2331_MOESM4_ESM.tif]

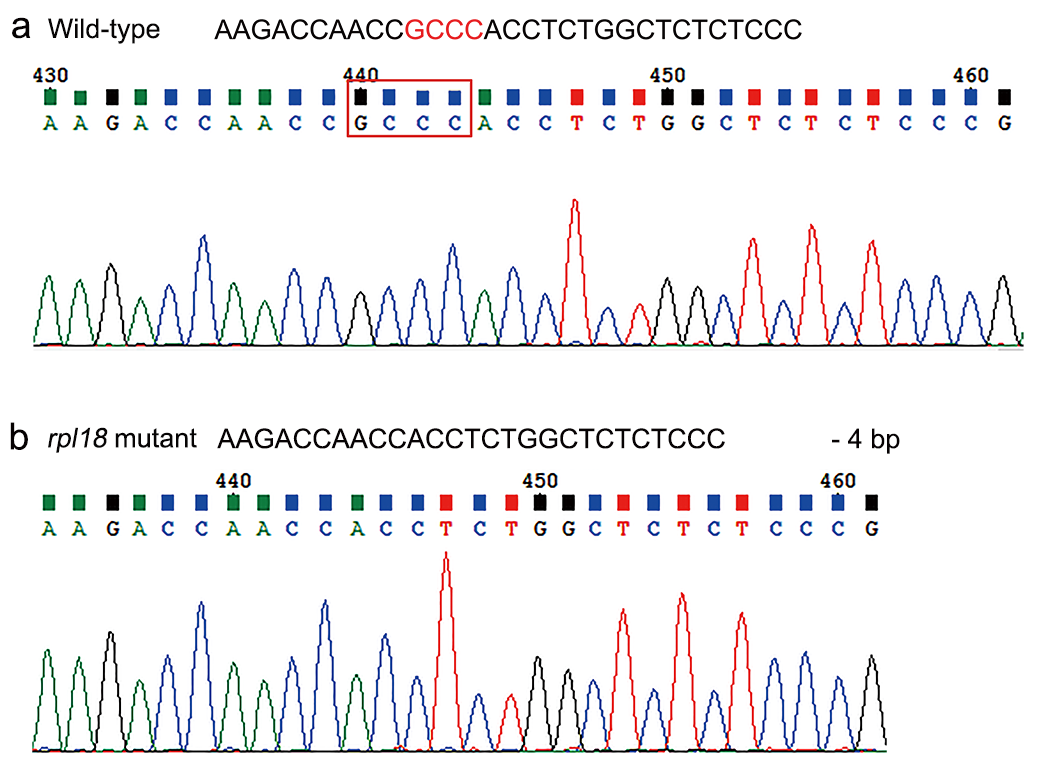

Supplement: Supplementary file 5 — Supplementary Figure 2 [file 41419_2020_2331_MOESM5_ESM.tif]

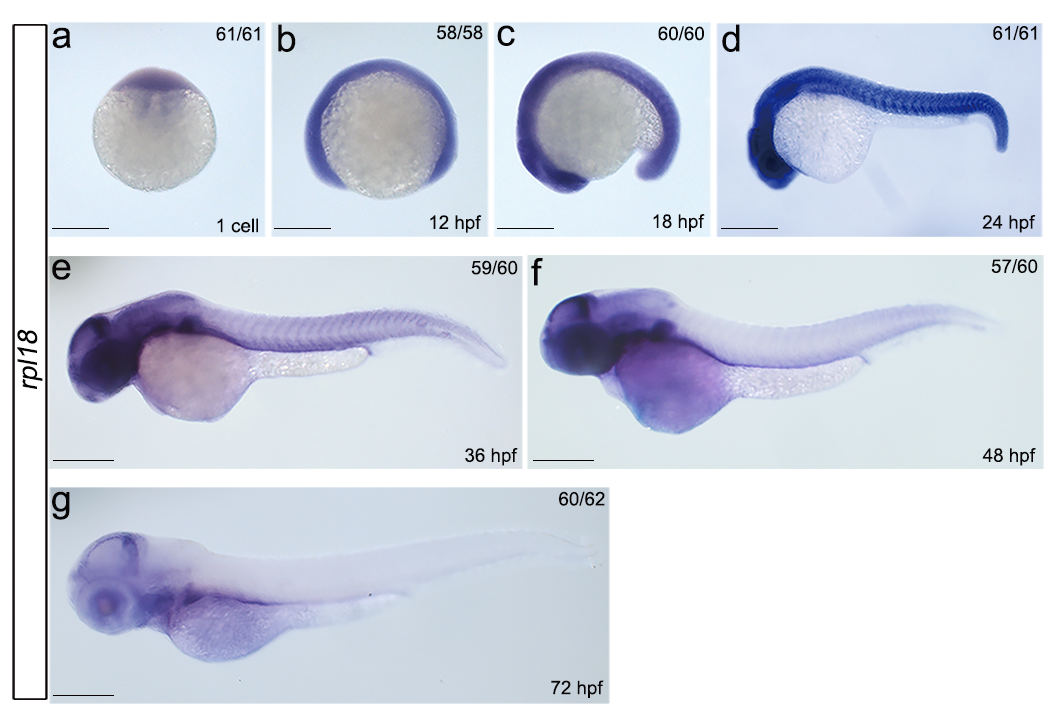

Supplement: Supplementary file 6 — Supplementary Figure 3 [file 41419_2020_2331_MOESM6_ESM.tif]

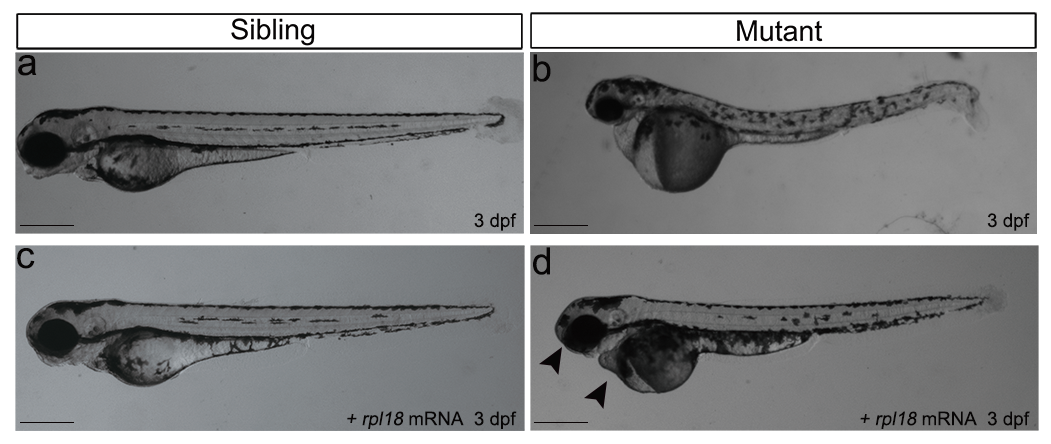

Supplement: Supplementary file 7 — Supplementary Figure 4 [file 41419_2020_2331_MOESM7_ESM.tif]

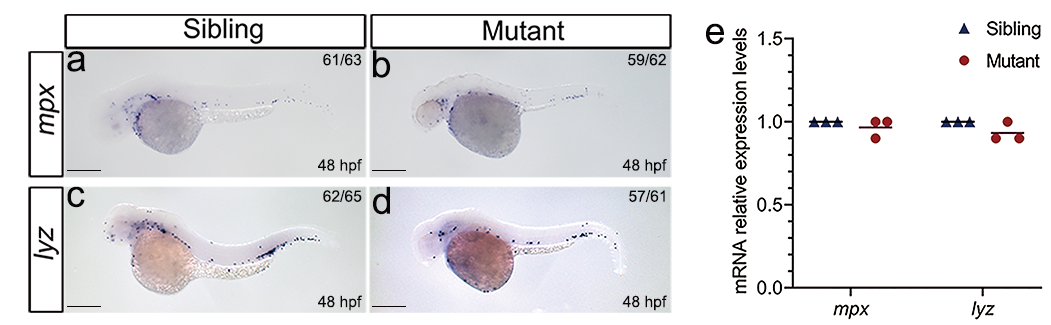

Supplement: Supplementary file 8 — Supplementary Figure 5 [file 41419_2020_2331_MOESM8_ESM.tif]

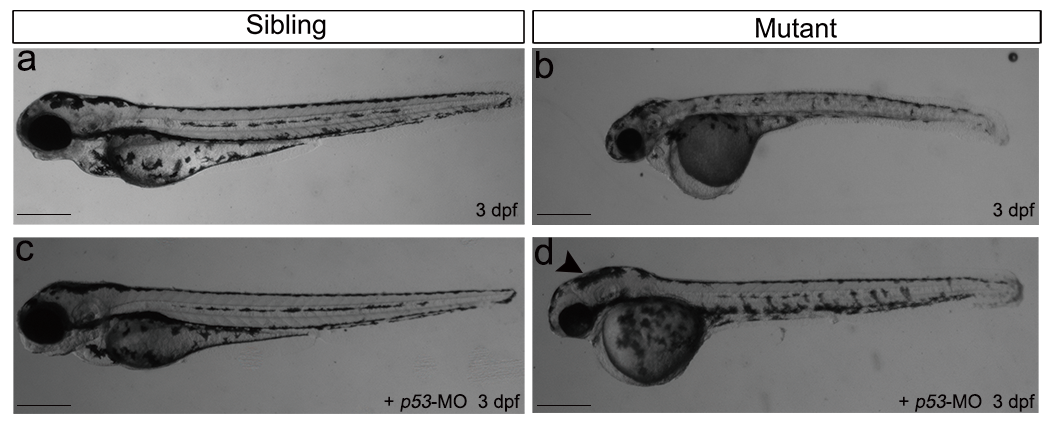

Supplement: Supplementary file 9 — Supplementary Figure 6 [file 41419_2020_2331_MOESM9_ESM.tif]

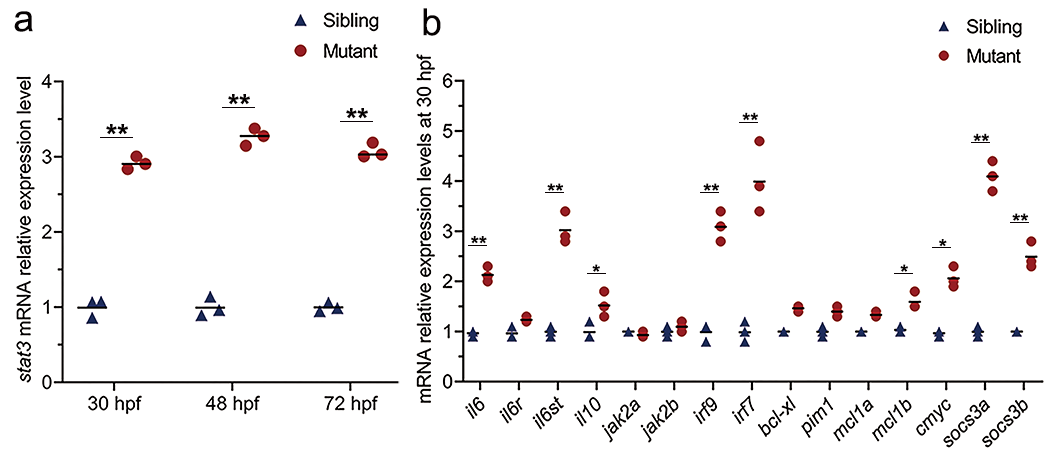

Supplement: Supplementary file 10 — Supplementary Figure 7 [file 41419_2020_2331_MOESM10_ESM.tif]

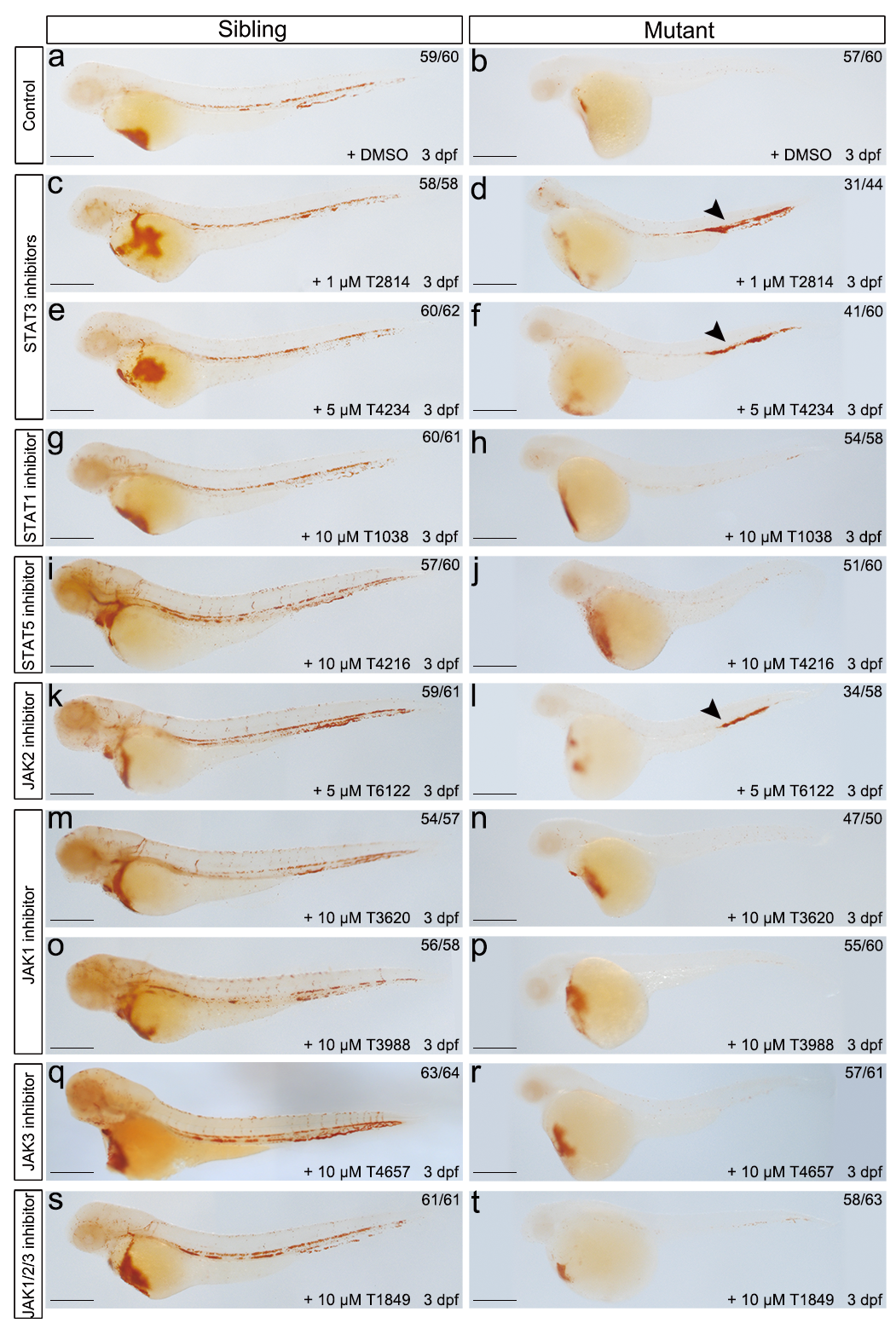

Supplement: Supplementary file 11 — Supplementary Figure 8 [file 41419_2020_2331_MOESM11_ESM.tif]

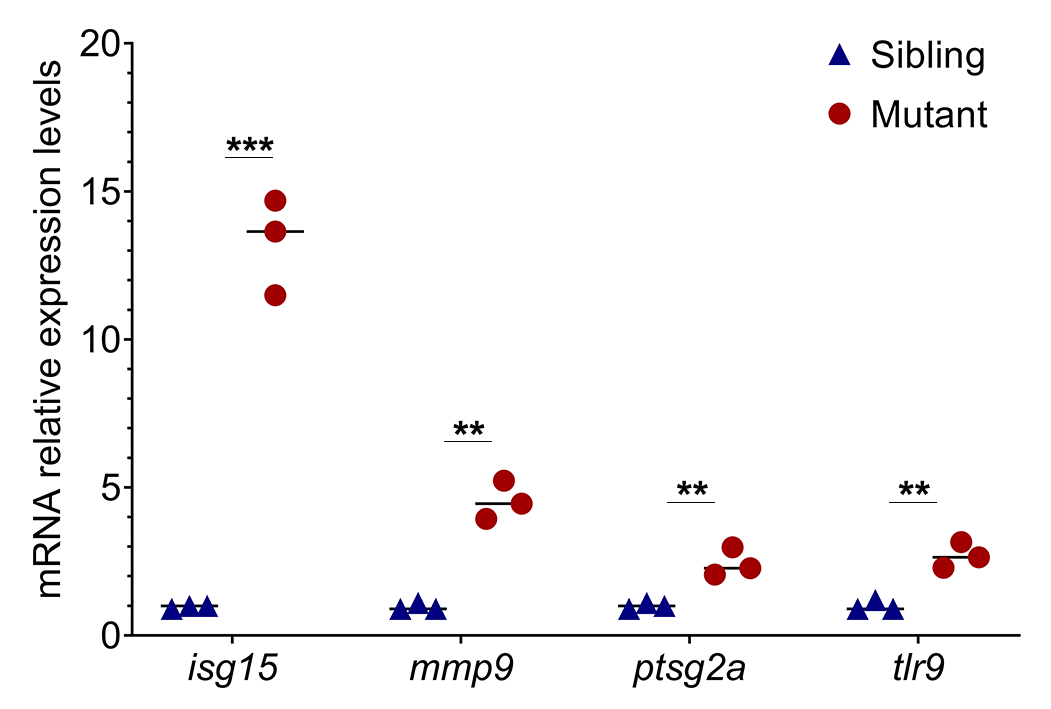

Supplement: Supplementary file 12 — Supplementary Figure 9 [file 41419_2020_2331_MOESM12_ESM.tif]

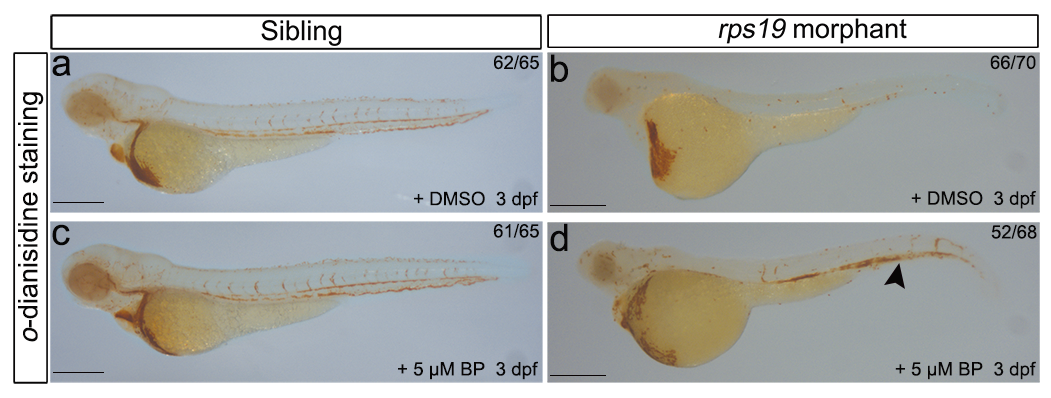

Supplement: Supplementary file 13 — Supplementary Figure 10 [file 41419_2020_2331_MOESM13_ESM.tif]

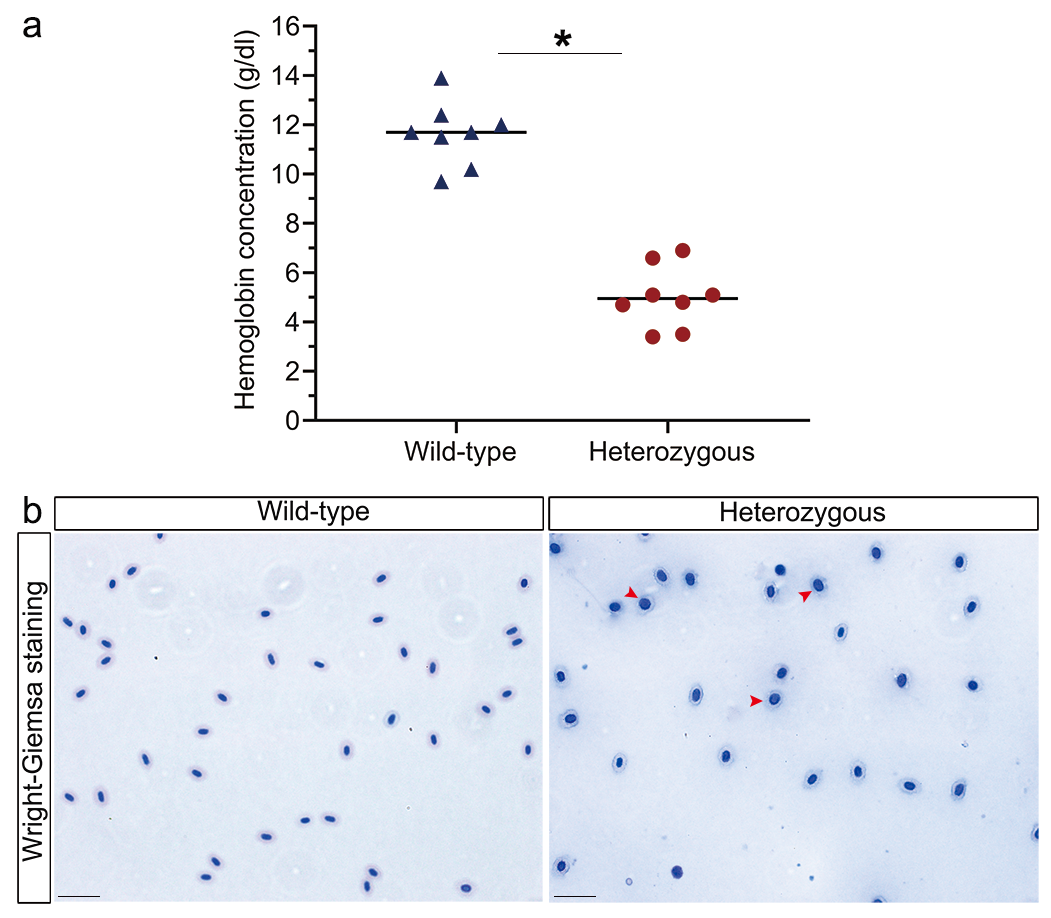

Supplement: Supplementary file 14 — Supplementary Figure 11 [file 41419_2020_2331_MOESM14_ESM.tif]

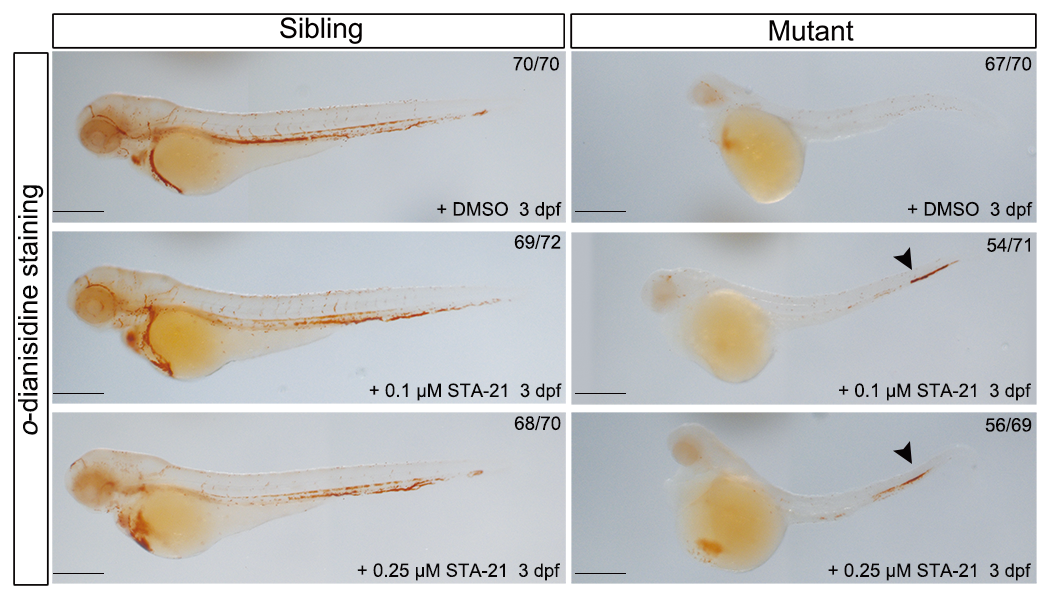

Supplement: Supplementary file 15 — Supplementary Figure 12 [file 41419_2020_2331_MOESM15_ESM.tif]
